# High-Pressure Balloon Dilatation in Infants with Primary Obstructive Megaureter: A Single-Center Case Series

**DOI:** 10.5152/tud.2025.24045

**Published:** 2025-06-04

**Authors:** Anna Suihko, Liisi Ripatti, Niklas Pakkasjärvi

**Affiliations:** 1 Department of Pediatric Surgery, Turku University Hospital, University of Turku, Turku, Finland; 2 Department of Surgery, University of Turku, Vaasa Central Hospital, Vaasa, Finland

**Keywords:** Primary obstructive megaureter, hydronephrosis, high-pressure balloon dilatation, infant, endoscopic, treatment

## Abstract

**Objective:**

The aim was to evaluate the efficacy of high-pressure balloon dilatation (HPBD) for primary obstructive megaureter (POM) treatment in infants.

**Methods:**

The authors retrospectively reviewed medical records of 5 infants diagnosed with symptomatic or progressive POM and treated with HPBD between 2015 and 2022 in one hospital, analyzing changes in ureteral and anteroposterior pelvic diameters, Society for Fetal Urology grading, parenchymal thickness, differential renal function, complications, and subsequent surgical needs.

**Results:**

High-pressure balloon dilatation was performed on 5 patients, median age 5 months. No statistically significant changes were detected in ureteral diameter (median 11.0-7.0 mm, *P* = .125), anteroposterior diameters (median 21.5-18 mm, *P* = .255), parenchymal thickness (median 5.0-5.0 mm, *P* = .317), or differential renal function post-procedure. Follow-up was median 34 months. Three patients showed improvement in obstructive renogram findings. Complications were primarily related to guidewire insertion and double-J stent placement. Two patients, both younger than 6 months, required open ureteral reimplantation.

**Conclusion:**

High-pressure balloon dilatation serves as a minimally invasive approach for POM but is not universally effective, with a high complication rate and 40% of infants needing open surgery post HPBD.

## Introduction

Primary obstructive megaureter (POM) is a congenital condition marked by ureteric dilation due to obstruction at the vesicoureteral junction (VUJ), accounting for up to 10% of prenatal hydronephrosis detected.^1^ Primary obstructive megaureter is most often seen in boys and on the left side. In approximately 70% of the cases, the obstruction is unilateral.^2^ Due to the evolution of prenatal ultrasound diagnostics, the age at diagnosis has significantly decreased, shifting the presentation from symptomatic cases to largely asymptomatic ones, with most patients being diagnosed antenatally.^3^ In most cases, the obstruction resolves spontaneously within the first months of life without compromising renal function, and no surgical treatment is needed.^4^ Although most POM cases can be managed conservatively, a debate remains over the mode, necessity, and timing of surgical intervention. Indications for surgical intervention include a continuous progression of hydroureteronephrosis coupled with recurring urinary tract infections (UTIs), as well as renal function deterioration or parenchymal reduction.

Historically, open ureteral reimplantation has been the standard of surgical treatment of POM. High complication rates and technical difficulties, especially complicating surgery in small infants,^5^ have raised interest in alternative strategies. These include initial diversion via cutaneous ureterostomy with a subsequent delayed definitive reimplantation, employing techniques such as refluxing end-to-side reimplant or mini-tapering,^6^^-^^10^ and exploring endoscopic methods. Endoscopic high-pressure balloon dilatation (HPBD) of the VUJ has become a viable primary treatment option for POM and promising results have been obtained in several studies: a recent systematic review showed a success rate of 71% after 1 HPBD and 79% after 2 HPBD.^11^ While HPBD seems appropriate for older children based on current systematic reviews,^11^,^12^ its application in infants remains largely underexplored. In addition, success rates for infants seem lower than for children over 12 months of age. In light of this, the authors embarked on a study to evaluate the outcomes of HPBD in this demographic.

In this retrospective study conducted between 2015 and 2022, the authors aimed to evaluate the outcomes and adverse effects of HPBD in infants with progressive or symptomatic POM. Specifically, the authors’ objectives included assessing the efficacy of HPBD in relieving obstruction, and its potential to obviate the need for more definitive open surgical interventions for patients for whom conservative management has been unsuccessful and are facing open reimplantation. Additionally, the authors sought to identify the incidence of postoperative complications associated with HPBD, graded according to Clavien-Dindo.^13^

## Material and Methods

The authors conducted a retrospective study on infants diagnosed with POM, who presented with symptomatic or progressive findings under the age of 1 year, treated by HPBD in Turku University Hospital during 2015-2022. The authors’ institution operates as a tertiary pediatric urology center, providing services to a region home to about 15% of the population. In 2023, a total of approximately 45 000 live births were reported in the country. During the study period, a total of 5 renal units were treated in the authors’ institution. Data were collected from hospital databases. This study represents the first report of HPBD outcomes in POM patients treated in the authors’ institution.

**Figure 1.**
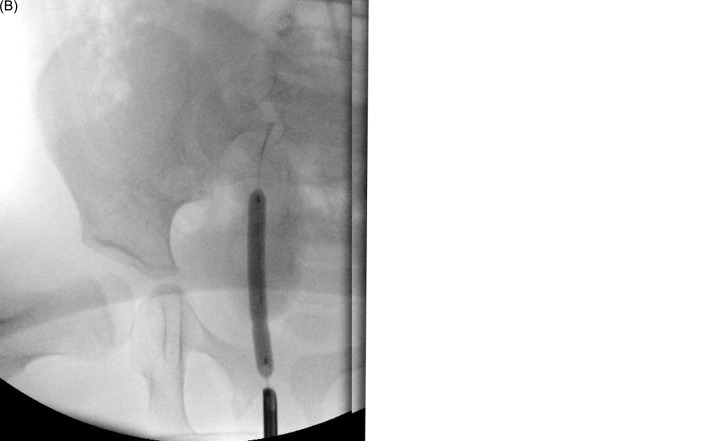
Intraoperative fluoroscopy of high-pressure balloon dilation of the obstructive vesicoureteral junction on the right side. The balloon is dilated until the stenotic waist (visible in 1A) disappears (1B).

The indications for performing HPBD were progressive hydroureteronephrosis with obstructive curve in MAG3, parenchymal thinning or differential renal function reduction of over 10% with or without UTI. The procedure was performed under general anesthesia using a CH 7.9 cystoscope. All patients received a single intravenous dose of antibiotic prophylaxis (cefuroxime 50 mg/kg) after anesthesia induction before the procedure. At first, antegrade pyelography was performed using a 3FR diametral ureteral catheter to confirm VUJ obstruction. Next, a 0.014 inch guidewire was passed through the ureteral catheter, the ureteral catheter removed, followed by advancement of the dilating PTA balloon over the guidewire. The diameter of the PTA catheter ranged from 2 mm to 5 mm and length from 20 mm to 40 mm. In the authors’ experience, shorter balloons are more challenging to maintain at the optimal position. The balloon was dilated until the stenotic waist disappeared under fluoroscopic imaging (see Figure 1A and B), required pressure ranging from 14 ATM to up to 20 ATM. Double-J stent was inserted and prophylactic antibiotic was administered during the stent treatment.

All patients underwent MAG-3 renography and ultrasound before and after the treatment. The measured outcome was the relief of obstruction and the decrease iin ureteral diameter and anteroposterior diameter of the renal pelvis. Success criteria for HPBD were defined as a decrease iin obstruction in diuretic renogram and a reduction in hydroureteronephrosis. Clinical data and complications were analyzed as well.

The outcomes analysis was conducted in Microsoft Excel®. The results are presented through means and medians. P-values were calculated with the Wilcoxon signed-rank test and *P* < .05 was considered statistically significant.

### Compliance with Ethical Standards

The study was approved by Turku University Hospital Clinical Research Center in Oct 18th 2022 (permission number T238/2022). This was a retrospective register study and thus no informed consent was required and the participants were not contacted. Legal basis for processing of personal data is public interest and scientific research (EU General Data Protection Regulation 2016/679 (GDPR), Article 6(1)(e) and Article 9(2)(j); Data Protection Act, Sections 4 and 6).

## Results

Five patients underwent HPBD during the study period. The indications for treatment included UTI in 3 patients and obstructive renograms with progressive ureteral dilation and parenchymal thinning or DRF reduction in all patients. Treatment distribution was equitable across affected sides: 3 patients received treatment on the left side, 2 on the right. One patient, presenting with bilateral obstruction, was first observed and experienced spontaneous resolution on the left side and thus required treatment solely on the right. Endoscopy was performed at a median age of 5 months (IQR 5-7), involving 4 male patients, constituting 80% of the cohort. The median duration of follow-up was 34 months (IQR 21-37). In 60% of the cases, the patients did not require further surgical interventions.

### Primary Outcomes: Efficacy

**Figure 2.**
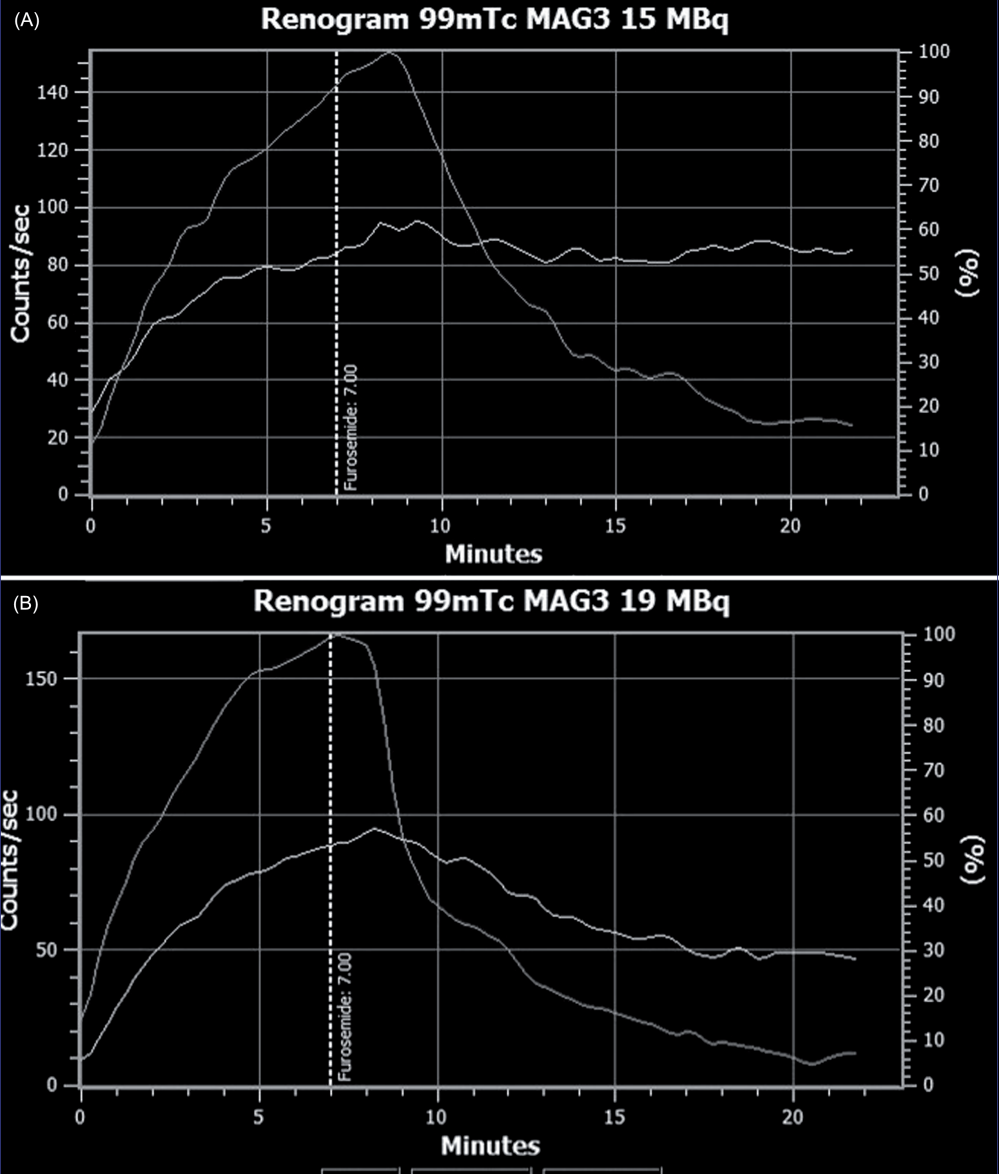
Renogram curves before (2A) and after (2B) high-pressure balloon dilatation.

Effectiveness of HPBD was gauged by changes in ureteral diameter, anteroposterior diameter of the renal pelvis, differential renal function as per MAG-3 renogram, and relief of obstruction in renogram. No statistical changes could be observed in ureteral diameter measurements pre- and postoperative (median 11.0 mm [IQR 10-15] to 7.0 mm [IQR 7-8], *P* = .125), AP diameters pre- and postoperative (median 21.5 mm [IQR 18-26] to 18 mm [IQR 12.5-25], *P* = .255), parenchymal thickness pre- and postoperative (median 5.0 mm [IQR 0] to 5.0 mm [IQR 0], *P* = .317), or differential renal function (median 44.0% [IQR 43-46] to 38 (IQR 33-49), *P* = .3125). Although no statistically significant changes could be detected, the progression of preoperative values was halted through the procedures. Obstructive findings in renograms were relieved in 3 patients (see Figure 2A and B), whereas 2 underwent subsequent open reimplantation.

Specific technical challenges arose in 2 cases: for patient 3, inserting the guidewire into the ureter cystoscopically proved impossible due to the tightness and crated-like morphology of the ureteric orifice. Thus, balloon dilatation was performed by a radiologist through an antegrade approach. Despite having performed the balloon dilatation, the ureteral orifice was still so tight that a double-J stent could not be advanced through it, necessitating the placement of a pyelostomy catheter. The pyelostomy catheter was later changed under general anesthesia due to UTI (Clavien-Dindo IIIb). A secondary dilatation attempt was also unsuccessful: the VUJ was still very tight and the guidewire could not be inserted cystoscopically and thus, reimplantation was performed under the same anesthesia. Patient 2 encountered difficulties with double-J stent insertion: after successful cystoscopic HPBD, the double-J stent was dislodged twice when the guidewire was removed. After this, the swelling in the VUJ increased so that the guidewire could no longer be inserted in the ureter cystoscopically and the stent was then placed antegrade by a radiologist under the same anesthesia. The further recovery of patient 2 was uneventful.

### Secondary Outcomes: Complications

Postoperative complications were stratified as follows: Clavien-Dindo grade I occurred in patient 1 who had macroscopic hematuria during double-J treatment. A grade II complication occurred in patient 5 who had UTI during double-J treatment 1 month after HPBD and the stent was removed slightly earlier than planned. In patient 3, the pyelostomy catheter had to be replaced under general anesthesia (grade IIIb complication) due to UTI. The same patient experienced mild hematuria and fever postoperatively unrelated to UTI and was diagnosed with roseola. Two patients (40%) experienced UTIs, one of which was associated with a double-J stent and the other with the pyelostomy catheter.

During follow-up, 2 patients (patient no. 1 and 3), both younger than 6 months at the time of initial treatment, required open ureteral reimplantation. The indications for proceeding to open ureteral reimplantation were increasing hydroureteronephrosis, an obstructive curve in the diuretic renogram, parenchymal reduction, and a very tight VUJ despite previous balloon dilatation(s). Histopathological examination of specimens from these reimplantations revealed inflammation and chronic fibrosis. Additionally, another patient underwent a second HPBD and remained symptom-free. No further surgical interventions were required in 40% of the cases.

Difficulty with double-J stent insertion was encountered in 2 cases. However, in the majority, balloon dilatation followed by stent placement could be performed. One stent dislodged spontaneously 1 week before planned removal, and the average stenting duration was 2.3 months (range 0.9-3.0 months) for the rest. Prophylactic antibiotics were administered post-procedure and discontinued after the stenting period in 60% of cases, while extended prophylaxis was deemed necessary in 40% for the 2 patients who had UTIs during stent and pyelostomy treatment.

In all cases, the mean hospital stay post-treatment was 1 day, underscoring the minimally invasive nature of HPBD and its postoperative management.

Four patients underwent preoperative screening for VUR: In 3 patients, VUR screening was performed using voiding cystourethrography. Two of these patients showed grade 1/5 VUR (1 during the voiding phase). In 1 patient, direct isotope cystography was used to rule out VUR.

Postoperatively only 1 patient underwent voiding cystourethrogram (VCUG), which showed no signs of VUR. Routine screening for the remaining patients was deemed unnecessary as they did not exhibit UTIs after stent or pyelostomy removal.

## Discussion

While endoscopic HPBD presents a less invasive alternative for managing POM, it does not eliminate the need for traditional open surgery in all infants. The endoscopic procedure was conducted at a median age of 5 months, predominantly in male patients, with a median follow-up of 34 months. Notably, 40% of the patients required no further surgical interventions. This suggests that while HPBD can effectively reduce the necessity for more invasive procedures in a significant subset of patients, it is not universally sufficient. The authors’ primary outcomes focused on the effectiveness of HPBD, measured by ureteral diameter, anteroposterior diameter of the renal pelvis, differential renal function, and relief of obstruction in diuretic renogram. Despite the absence of statistically significant changes in these parameters, the progression of preoperative values was effectively halted, and obstructive findings in renograms were relieved in 60% of cases.

Megaureter predominantly affects boys and is more commonly observed on the left side, with around 30% of cases presenting bilateral obstruction.^2^ The underlying pathophysiology of POM is often attributed to abnormal or delayed muscular development in the distal ureter, resulting in a functional obstruction due to impaired peristalsis. While the majority of POM cases spontaneously resolve in the early months of life, approximately 10%-25% require surgical intervention to prevent renal function deterioration or manage symptoms such as recurrent UTIs, pyelonephritis, hematuria, calculi, or persistent flank pain.^2^,^4^,^14^ The authors opted to only treat symptomatic and/or progressive patients in this series. Unfortunately, the authors do not have data on all patients followed in the authors’ center for POM since most have spontaneously healed and have been recorded with diagnosis code for antenatal hydronephrosis.

Open ureteral reimplantation has traditionally been the cornerstone of surgical management for POM, presenting a high success rate. In a study involving children with an average age of 4.9 years, the success rate of open ureteral reimplantation surgery for POM stood at 82%.^15^ However, concerns over its associated morbidity^16^,^17^ and the risk of reoperation have prompted the exploration of alternative surgical methods. Particularly in infants, the challenges of integrating a dilated ureter into a small bladder raise concerns, with early intervention potentially leading to subsequent bladder dysfunction. Alternatives have thus gained traction, ranging from initial cutaneous ureterostomy for temporary diversion followed by later definitive reimplantation to innovative techniques like refluxing end-to-side reimplant or mini-tapering.^6^^-10^ Additionally, endoscopic strategies are being explored to mitigate these concerns, marking a shift toward less invasive surgical management for POM.

The introduction of endoscopic HPBD as a primary treatment for children with POM by Angulo et al^18^ in 2007 marked a significant shift toward less-invasive approaches. Subsequent studies have validated HPBD as a safe, minimally invasive, and effective treatment, offering a compelling alternative to traditional open ureteral reimplantation.^11^ Despite a complication rate of 33%, including postoperative infections and vesicoureteral reflux in 12% and 7.8% of patients, respectively, HPBD remains a viable option.^11^ However, the dearth of studies on HPBD in infants underscores the need for further research to evaluate its efficacy in this demographic. Building on these insights, the authors’ study further corroborates the limited series in infants, where HPBD stands as an effective treatment modality for POM, reinforcing its utility and safety across various patient demographics. High-pressure balloon dilatation minimizes the risk of permanent bladder function damage and does not preclude the option of future open surgery, as it preserves the bladder wall and ureteral circulation. High-pressure balloon dilatation has emerged as a promising primary or definitive treatment strategy for POM by potentially delaying or even obviating the need for more invasive procedures. However, as highlighted here, some patients will continue to require further open surgery.

Several studies have reported a success rate of 79%-90% after the first HPBD.^19^,^20^ The success rate in the authors’ series was 40% after the first HPBD and 60% after secondary HPBD. In 2 cases, unsuccessful attempts of balloon dilatation were followed by ureteral reimplantation. Both children who underwent further ureteral reimplantation were under 6 months (mean 3-5 months, range 2-5 months) during the initial endoscopic approach. Mean age at the time of open ureteral reimplantation was 10 months (range 7-13 months), with a mean of 6-5 months (range 2-11 months) after the primary endoscopic treatment. In recent studies, HPBD has shown similar results in treating infants as older children: a recent systematic review by Skott et al^12^ reported a success rate of 61.9% on infants under 12 months of age and 71.8% on older children with an overall rate of 14.3% requiring subsequent reimplantation. The scarcity of reports focusing specifically on infant patients presents a significant gap in the literature. Boswell et al^21^ highlight this gap with their study demonstrating an 80% success rate for HPBD in treating symptomatic POM in a cohort of fifteen infants with a median age of 7.6 months. Similarly, Torino et al^22^ have documented sustained positive outcomes over a 2-year follow-up period in an earlier series of infants. Capozza et al^23^ reported an 83% success rate in a cohort of 12 infants. In contrast, the success rate in our series was somewhat lower. This discrepancy might be partly attributable to the initial learning curve and anatomical variations, which posed specific challenges such as difficulties with guidewire insertion and double-J stent placement, leading to alternative surgical interventions and, in one case, ureteral reimplantation. Although HPBD effectively alleviates obstruction within the distal ureteral segment, deficient peristalsis may persist. This phenomenon could explain the curative success of open ureteral re-implantation in patients for whom HPBD is unsuccessful, as it involves excision of the non-peristaltic distal segment. This hypothesis is supported by histopathological findings of inflammation and fibrosis in the excised segments from the 2 patients in whom HPBD was unsuccessful in the authors’ cohort. The postoperative complications the authors observed were generally mild to moderate, including a singular intraoperative complication classified as Clavien-Dindo grade IIIb and minor postoperative issues like hematuria, and fever. These were either self-resolving or effectively managed with treatment.

Our findings also underscore the minimally invasive nature of HPBD, evidenced by the short mean hospital stay and absence of postoperative vesicoureteral reflux across all patients. This suggests that HPBD represents a viable treatment strategy for obstructive uropathy in infants, offering an effective balance between therapeutic efficacy and minimal invasiveness. Future research endeavors must refine patient selection criteria and optimize treatment protocols, thus enhancing outcomes and reducing the necessity for further surgical interventions.

The observed complications in the authors’ patient cohort align with those previously reported, predominantly UTIs, hematuria, and challenges related to guidewire insertion. Ripatti et al^11^ reported a complication rate of 33%, predominantly mild to moderate, necessitating further intervention under general anesthesia in a subset of cases. In the authors’ series, the incidence of UTI post-first endoscopic treatment stood at 40%, and 60% in all patients, while none of the patients presented with pyelonephritis in long-term post-treatment.

The primary limitations of the authors’ study include its small sample size and the lack of a comparative control group, constraints that limit the generalizability of the authors’ findings and the authors’ ability to draw definitive conclusions. Additionally, routine postoperative VCUG was not performed, which may have led to underdetection of asymptomatic VUR. While this approach was based on the absence of clinical symptoms and ultrasound during follow-up, the potential role of asymptomatic VUR remains debated and may warrant routine assessment, as highlighted in recent reviews.^24^ This highlights the necessity for further, more expansive research to fully ascertain the efficacy and safety profile of HPBD in treating POM in infants.

In conclusion, endoscopic HPBD is a minimally invasive approach for POM, yet it is not universally sufficient, as evidenced by the high complication rate and 40% of the authors’ patients requiring open surgery after HPBD. This limitation may be most notable in patients with a non-peristaltic distal ureter, for whom open ureteral reimplantation—removing the affected segment—offers a definitive solution. Thus, while HPBD reduces invasive surgeries in many cases, its effectiveness is dependent on patient-related conditions, necessitating the development of a more tailored treatment strategy for the treatment of POM.

## Data Availability

The data that support the findings of this study are not available on request from the corresponding author.
